# Dioxin Exposure and Age of Pubertal Onset among Russian Boys

**DOI:** 10.1289/ehp.1003102

**Published:** 2011-04-28

**Authors:** Susan A. Korrick, Mary M. Lee, Paige L. Williams, Oleg Sergeyev, Jane S. Burns, Donald G. Patterson, Wayman E. Turner, Larry L. Needham, Larisa Altshul, Boris Revich, Russ Hauser

**Affiliations:** 1 Department of Environmental Health, Harvard School of Public Health, Boston, Massachusetts, USA; 2 Channing Laboratory, Department of Medicine, Brigham and Women’s Hospital, Harvard Medical School, Boston, Massachusetts, USA; 3 Pediatric Endocrine Division, Departments of Pediatrics and Cell Biology, University of Massachusetts Medical School, Worcester, Massachusetts, USA; 4 Department of Biostatistics, Harvard School of Public Health, Boston, Massachusetts, USA; 5 Samara State Medical University, Department of Physical Education and Health, Samara, Russia; 6 Chapaevsk Medical Association, Chapaevsk, Russia; 7 Centers for Disease Control and Prevention, Atlanta, Georgia, USA; 8 Axys Analytical Solutions, Sidney, British Columbia, Canada; 9 Fluid Management Systems (FMS), Boston, Massachusetts, USA; 10 Exponent, Inc., Maynard, Massachusetts, USA; 11 EnviroSolutions Consulting, Inc., Auburn, Georgia, USA; 12 Centers for Disease Control and Prevention Foundation, Atlanta, Georgia, USA; 13 Environmental Health and Engineering, Inc., Needham, Massachusetts, USA; 14 Center for Demography and Human Ecology of the Institute of Forecasting, Russian Academy of Sciences, Moscow, Russia; 15 Vincent Memorial Obstetrics and Gynecology Service, Andrology Laboratory and In vitro Fertilization Unit, Massachusetts General Hospital, Harvard Medical School, Boston, Massachusetts, USA

**Keywords:** children, dioxins, furans, growth, PCBs, polychlorinated biphenyls, pubertal stage, puberty, testicular volume

## Abstract

Background: Animal data demonstrate associations of dioxin, furan, and polychlorinated biphenyl (PCB) exposures with altered male gonadal maturation. It is unclear whether these associations apply to human populations.

Objectives: We investigated the association of dioxins, furans, PCBs, and corresponding toxic equivalent (TEQ) concentrations with pubertal onset among boys in a dioxin-contaminated region.

Methods: Between 2003 and 2005, 499 boys 8–9 years of age were enrolled in a longitudinal study in Chapaevsk, Russia. Pubertal onset [stage 2 or higher for genitalia (G2+) or testicular volume (TV) > 3 mL] was assessed annually between ages 8 and 12 years. Serum levels at enrollment were analyzed by the Centers for Disease Control and Prevention, Atlanta, Georgia, USA. We used Cox proportional hazards models to assess age at pubertal onset as a function of exposure adjusted for potential confounders. We conducted sensitivity analyses excluding boys with pubertal onset at enrollment.

Results: The median (range) total serum TEQ concentration was 21 (4–175) pg/g lipid, approximately three times higher than values in European children. At enrollment, boys were generally healthy and normal weight (mean body mass index, 15.9 kg/m^2^), with 30% having entered puberty by G2+ and 14% by TV criteria. Higher dioxin TEQs were associated with later pubertal onset by TV (hazard ratio = 0.68, 95% confidence interval, 0.49–0.95 for the highest compared with the lowest quartile). Similar associations were observed for 2,3,7,8-tetrachlorodibenzo-*p*-dioxin and dioxin concentrations for TV but not G2+. Results were robust to sensitivity analyses.

Conclusions: Findings support an association of higher peripubertal serum dioxin TEQs and concentrations with later male pubertal onset reflected in delayed testicular maturation.

The transition from prepuberty to sexual maturity entails rapid physical, hormonal, and behavioral development. Alterations in the timing of pubertal onset or pace of its progression can adversely affect not only physical and sexual maturation, but also social, cognitive, and behavioral development and adult health (Graber et al. 2004; Michaud et al. 2006). For example, earlier puberty incurs risk for metabolic syndrome and obesity in later life, and delayed puberty is associated with decreased bone mineral density in adults, raising concerns about increased fracture risk (Biro et al. 2003; Finkelstein et al. 1996; Van Lenthe et al. 1996).

In recent decades, suggestive evidence of earlier onset of breast development and age at menarche has been observed in girls, but data in boys are limited (Biro et al. 2010; Euling et al. 2008; Herman-Giddens et al. 1997, 2001; Sorensen et al. 2010). Explanations for this possible trend include changes in diet and activity and/or environmental exposures. Chemicals that can disrupt gonadal steroidogenesis and neuroendocrine pathways, such as organochlorine pollutants including dioxins, furans, and polychlorinated biphenyls (PCBs), are of particular concern (Jacobson-Dickman and Lee 2009; Schoeters et al. 2008). Despite efforts to limit dioxin emissions, and longstanding bans on PCB manufacture and use, human exposure is ongoing, primarily through diet. For example, fish and dairy are potential exposure sources because of organochlorines’ lipophilic properties of, long half-lives (years to decades), and propensity to bioconcentrate (Schecter et al. 2001).

Gestational or lactational dioxin exposures in animals have been consistently associated with delayed male pubertal onset (Bell et al. 2007; Hamm et al. 2003; Theobald et al. 2003). However, the few human epidemiologic studies of organochlorine exposures and puberty in boys did not examine onset and had inconsistent findings. Where direct exposure biomarkers were used, dioxins, furans, or PCBs were associated with no differences in (Den Hond et al. 2002; Gladen et al. 2000; Leijs et al. 2008) or earlier (Den Hond et al. 2010) male genital maturation among older adolescents, but inferences were often limited by small sample sizes (Den Hond et al. 2002; Leijs et al. 2008).

Chapaevsk, Russia, is an industrial town contaminated with dioxins consequent to past production of chemical warfare agents and recent production of chlorinated chemicals. Waste from these industries resulted in pervasive dioxin contamination of soils, water, and local food (Revich et al. 1999; Sergeyev et al. 2007). Community concerns regarding potential health hazards from this contamination led to a study of dioxins and male pubertal development.

## Materials and Methods

*Study population.* The Chapaevsk study is an ongoing prospective study of 499 generally healthy boys (Hauser et al. 2008). All male residents 8–9 years of age (*n* = 623) were identified between 2003 and 2005 from health insurance records and the town’s clinic system. Of these, 572 met eligibility criteria and 516 (90%) agreed to participate. Children were ineligible if their address was unavailable, if they were likely to move during the study, or if they had severe cerebral palsy. After enrollment, 17 children living in orphanages were excluded because of missing birth or family history. For this analysis, 10 additional boys were excluded for chronic illnesses that could affect growth (e.g., severe asthma or malignancy), leaving 489 boys.

Once enrolled, each boy underwent a physical examination, provided a blood sample, and together with his mother or guardian completed health, lifestyle, and dietary questionnaires. Annual follow up examinations were conducted on or close to each boy’s birthday and questionnaires were updated. For this analysis, 3 years of follow-up data were available, with each boy observed up to four times between ages 8–11 or 9–12 years.

The study was approved by the Human Studies Institutional Review Boards of the Chapaevsk Medical Association (Chapaevsk, Russia); Harvard School of Public Health and Brigham and Women’s Hospital (Boston, MA, USA), and University of Massachusetts Medical School (Worcester, MA, USA). The parent or guardian signed an informed consent, and each boy signed an assent before participation.

*Growth and pubertal assessment.* At annual visits, an endocrinologist (O.S., with a nurse present) conducted standardized physical examinations without knowledge of the boy’s exposure. Examination included measurement of height in stocking feet (to the nearest 0.1 cm) using a fixed arm stadiometer and weight in underclothes (to the nearest 100 gm) using a balance scale. Body mass index (BMI; kilograms per square meter) was calculated from measured height and weight. Pubertal maturation was graded from 1 to 5 by visual inspection according to established criteria (Tanner and Whitehouse 1976). Testicular volume (TV) was measured using an orchidometer. Pubertal onset was defined as stage 2 or higher for genitalia (G2+) or TV > 3 mL for either testis.

*Questionnaire assessment.* At enrollment, each mother or guardian completed a nurse-administered questionnaire ascertaining *a*) the child’s birth and medical history, breast-feeding status, physical activity, *b*) family demographics, income, residential history, and *c*) parental reproductive and medical history, occupation, education, smoking, and alcohol consumption. Birth weight and gestational age were obtained from medical record review. Diet was ascertained using a food frequency questionnaire modified from a validated Russian Institute of Nutrition instrument (Burns et al. 2009; Martinchik et al. 1998).

*Organochlorine exposure assessment.* Fasting blood samples were collected before baseline examination, and the serum fraction was stored at –35°C until shipment for analysis at the National Center for Environmental Health at the Centers for Disease Control and Prevention (Atlanta, GA, USA). Analytes included 7 polychlorinated dibenzo-*p*-dioxins (PCDDs, or dioxins), 10 polychlorinated dibenzofurans (PCDFs, or furans), 4 co-planar PCBs (co-PCBs), 6 mono-*ortho*–substituted PCBs, and 31 other PCBs (non-dioxin-like PCBs) described in Burns et al. (2009).

For dioxin-like analytes, sera, method blanks, and quality control samples (aliquots of pooled bovine sera) were spiked with a mixture of ^13^C_12_-labeled PCDDs/PCDFs and co-PCBs as internal standards, and serum analytes were isolated by solid phase extraction (SPE) followed by a multicolumn automated cleanup and enrichment procedure (Turner et al. 1997). Analytes were separated on a DB-5 MS capillary column (Phenomenex, Torrance, CA, USA) and quantified using selected-ion-monitoring (SIM) high-resolution (10,000 resolving power) mass spectrometry (HRGC-ID/HRMS; Thermo Electron North America, LLC, West Palm Beach, FL, USA) (Patterson et al. 1987). Quantification by isotope dilution MS used calibration standards containing ^13^C_12_-labeled and unlabeled analytes.

A similar approach was used for mono-ortho and non-dioxin-like PCBs (Barr et al. 2003). Samples were spiked with ^13^C_12_-labeled PCBs, extracted by either large (Turner et al. 1997) or small (Sjodin et al. 2004) volume SPE, and analyzed using HR GC/MS in SIM (Barr et al. 2003).

For all analytes, quality control sample coefficients of variation combining between-run and within-run reproducibility were generally < 15%. All concentrations were expressed on a per-lipid basis, with serum total cholesterol and triglycerides measured enzymatically, and total lipids were calculated using the Phillips equation (Phillips et al. 1989). Congener concentrations below the limit of detection (LOD) were assigned the LOD divided by the square root of 2.

*Statistical analysis.* Dioxin toxic equivalents (TEQs) were computed on a lipid basis using the 2005 World Health Organization (WHO) toxic equivalency factors to weight the potency of each congener relative to 2,3,7,8-tetrachlorodibenzo-*p*-dioxin (TCDD) before summation (Van den Berg et al. 2006). Nine different exposure measures were considered: (1) total (summed) TEQ measures (picograms per gram lipid) for combined dioxin, furan, co-PCB, and mono-*ortho* PCB congeners; (2) TCDD (picograms per gram lipid); (3–5) total (summed) TEQs (picograms per gram lipid) for each of the dioxins, furans, and co-PCBs; (6–8) total (summed) concentrations (picograms per gram lipid) for each of the dioxins, furans, and co-PCBs; and (9) total (summed) concentrations of non-co-planar PCBs, including mono-*ortho*–substituted PCBs (ΣPCBs) (nanograms per gram lipid). Organochlorine measures were categorized into quartiles because of potential nonlinear associations. Analyses were repeated using a quartile indicator (1, 2, 3, 4) for exposure to test for trend across quartiles. Statistical significance was defined as a *p*-value < 0.05.

We used standard Cox proportional hazards models to assess time to pubertal onset as a function of exposure adjusted for potential confounders. Age of pubertal onset was assigned to the midpoint between age at the previous visit and age at the visit at which onset was noted. For boys in puberty at study enrollment (*n* = 141 by G2+; *n* = 66 by TV > 3 mL), age at onset was defined as 6 months before age at enrollment. Observations were censored at the last visit for boys not yet in puberty.

Sensitivity analyses were performed using both interval-censored likelihood-based models and repeated measures generalized estimating equation (GEE) models. The interval-censored approach does not assign a specific time of onset, but instead assumes that pubertal onset occurred in the interval between study visits. This approach was used to estimate overall mean age of pubertal onset, assuming a normally distributed age at onset, and mean age at pubertal onset for each exposure quartile, adjusted for confounders. The GEE approach was used to fit a logistic regression model for pubertal onset at each visit as a function of age at visit, with adjustment for potential confounders and correlation among multiple visits via an autoregressive structure. GEEs were also used to evaluate the impact of clustering within household for twins (four pairs) and siblings (three pairs). To account for possible examiner and laboratory drift over time, uncertainty regarding age of pubertal onset, and the potential for reverse causation (due to dilution of dioxin concentrations in larger, more mature boys), additional sensitivity analyses were performed excluding boys with pubertal onset at study entry and adjusting for year of organochlorine analysis.

Covariates considered in models included potential determinants of pubertal onset: age of child at examination, birth weight, gestational age, breast-feeding; nutrition, height, weight, and BMI at enrollment; household income; maternal age at birth and parity; prenatal smoking (active and secondhand) and alcohol intake; parental education; and blood lead (Williams et al. 2010). A core model was developed by first assessing the univariate relation of covariates to each pubertal onset measure and retaining those with a *p*-value < 0.20. Covariates meeting this criterion were included in a full model; backward selection (likelihood ratio test) was then used to iteratively exclude the least important covariates (retain *p* < 0.15). Covariates were retained if they were significant for at least one pubertal onset measure or if they resulted in a ≥ 10% change in exposure effect estimates when added, one at a time, into our final model. Because height and BMI at enrollment may be proxies for pubertal onset or on the causal pathway relating dioxins with onset, we performed sensitivity analyses excluding these covariates from the final model. Because age of mother at menarche was missing for 8% of participants, this covariate was added to the final models in sensitivity analyses.

The association of pubertal onset with each of the nine different exposure measures was assessed, one exposure at a time. These nine measures were moderately to strongly correlated (Spearman *r* = 0.44–0.90); therefore, secondary analyses were performed to assess the independent relation of dioxin-like versus non-dioxin-like exposures with pubertal onset. Specifically, final models for the relation of each dioxin-like measure (total TEQ, TCDD, as well as dioxin, furan, and co-PCB TEQ and concentration measures) with pubertal onset were rerun with non-co-planar PCB concentrations (ΣPCBs) added to the models.

## Results

*Demographic and exposure characteristics.* The boys were racially homogeneous (all Caucasian) and most were full term with normal birth weight and enrollment BMI (Table 1). Of the 489 boys, 473 had dioxin/furan measures and 468 had PCB measures. The median (range) total serum TEQs at 8–9 years of age was 21.1 (4.0–174.7) pg/g lipid, about three times higher than levels among European children of similar age (Table 2) (Leijs et al. 2008; Link et al. 2005).

**Table 1:** Demographic, physical examination, and family characteristics at study entry for Chapaevsk boys with dioxin or PCB levels (*n* = 473).

Characteristic	Missing (*n*)	*n* (%)	Mean (range)
Growth measures						
Age (years)		0				8.4 (7.8–9.4)
Height (cm)		0				130 (111–147)
Weight (kg)		0				27.1 (15.4–49.4)
BMI (kg/m^2^)		0				15.9 (11.8–25.2)
Overweight*a*		0		80 (17)		
Underweight*a*		0		28 (6)		
Pubertal maturation						
Tanner G2+		0		141 (30)		
TV > 3 mL		4		66 (14)		
Nutrition						
Calories/day (kcal)		3				2,820 (884–5,000)
Percent protein		3				11.6 (6.6–18.8)
Percent fat		3				33.9 (15.3–51.5)
Percent carbohydrate		3				54.5 (33.2–72.7)
Birth and neonatal history						
Birth weight (kg)		3				3.34 (1.40–4.80)
Low birth weight (< 2,500 g)		3		24 (5)		
Gestational age (weeks)		4				39.1 (30.0–44.0)
Preterm (< 37 weeks)		4		37 (8)		
Duration breast-fed (weeks)		11				27.4 (0–312.0)
Maternal characteristics and pregnancy exposures						
Age at birth (years)		5				23.9 (15.1–42.6)
Age at menarche (years)		39				13.3 (13.0–17.0)
Nulliparous		18		303 (67)		
Maternal pregnancy smoking		13		36 (8)		
Any household smoking		9		224 (48)		
Maternal pregnancy alcohol		16		59 (13)		
Household characteristics						
Family income [per month (US$)]		1				
< $175				164 (35)		
$175–$250				123 (26)		
> $250				185 (39)		
Parental education*b*		4				
≤ High school				37 (8)		
Some college or junior college				279 (59)		
College graduate				153 (33)		
Other exposures						
Blood lead ≥ 5 μg/dL		0		132 (28)		
**a**Overweight (≥ 1 SD above mean BMI for age); underweight (≥ 2 SD below mean BMI for age) using WHO standards (de Onis et al. 2007). **b**Maximum of mother’s and father’s education.						

**Table 2:** Serum dioxin, furan, and PCB TEQs (picograms TEQ per gram lipid), and dioxin, furan, and PCB concentrations among Chapaevsk boys at study enrollment (*n* = 473).

Organochlorine	Mean ± SD	25th	50th	75th	Maximum
TEQs (pg TEQ/g lipid)										
TCDD*a*		3.1 ± 3.1		1.3		2.8		3.9		44.9
PCDD TEQ		10.6 ± 9.5		4.5		8.2		13.6		89.8
PCDF TEQ		7.0 ± 11.2		3.0		4.2		6.9		154.3
Co-PCB TEQ*b*		8.1 ± 6.5		4.5		6.4		9.4		67.2
Total TEQ*c*		27.7 ± 22.0		14.4		21.1		33.2		174.7
Concentration (pg/g lipid)										
PCDD		160 ± 110		96		136		188		1,237
PCDF		57 ± 78		27		39		57		1,083
Co-PCB*d*		209 ± 143		129		181		246		2,067
Concentration (ng/g lipid)										
ΣPCBs*e*		331 ± 312		164		250		394		4,248
25th, 50th, and 75th are percentiles. **a**The median LOD for TCDD was 0.60 pg/g lipid; 123 (26%) of TCDD values were less than this LOD (Burns et al. 2009). **b**Sum of co-planar PCB TEQs [International Union of Pure and Applied Chemistry (IUPAC) congeners: 77, 81, 126, 169]. **c**Sum of TEQ measures for combined dioxin, furan, co-PCB and mono-ortho PCB congeners. **d**Sum of coplanar PCB concentrations (IUPAC congeners: 77, 81, 126, 169). **e***n* = 468 (IUPAC congeners: 18, 28, 52, 49, 44, 74, 66, 101, 99, 87, 110, 118, 105, 151, 149, 146, 153, 138/158, 128, 167, 156, 157, 178, 187, 183, 177, 172, 180, 170, 189, 201, 196/203, 195, 194, 206).										

*Pubertal onset characteristics.* Most (85%) boys had four annual examinations between 8–11 or 9–12 years of age, with 6, 4, and 5%, respectively, examined three times, twice, or once. At study entry (both 8- and 9-year-olds), 30% had entered puberty by G2+ and 14% by TV criteria. By 12 years of age, most had entered puberty (92% by G2+ and 83% by TV criteria). Overall, the estimated mean [95% confidence interval (CI)] age of onset by G2+ and TV, based on interval-censored models, was 9.4 (9.2–9.6) and 10.5 (10.3–10.7) years, respectively. In multivariable models (results not shown), pubertal onset was significantly earlier with higher birth weight (both measures), lower gestational age (G2+), higher percentage of dietary fat (TV), and greater height or BMI at the initial study visit (both measures). Conversely, pubertal onset was significantly later with maternal alcohol consumption during pregnancy (TV), low household income (TV), older maternal age at menarche (both measures), and high blood lead (both measures).

**Figure 1:**
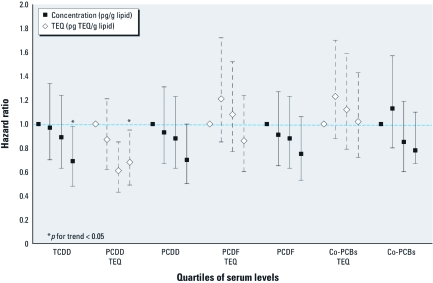
Adjusted HRs for pubertal onset according to quartiles of serum levels of TCDD, dioxins, furans, and co-planar PCBs. HRs with bars denoting 95% CIs for the association of serum quartiles of both concentrations and TEQs for dioxin (TCDD), dioxins (PCDD), furans (PCDF), and co-PCBs with risk of pubertal onset (TV > 3 mL) between ages 8 and 12 years among Chapaevsk boys. Results adjusted for birth weight, gestational age, parental education, household income, diet at 8 or 9 years old (total calories, percent protein, percent fat), blood lead ≥ 5 μg/dL, maternal pregnancy alcohol intake, baseline height, and BMI.

*Association of dioxins, furans, and PCBs with pubertal onset.* In multivariable Cox proportional hazards models, pubertal onset was later with increasing dioxin exposure for TV but not G2+ (Table 3; Figure 1). For example, for TV > 3 mL, the hazard ratio (HR) was 0.69 (95% CI, 0.48– 0.98) for the highest compared with the lowest quartile of serum TCDD. Similar associations were observed for PCDD TEQs (Table 3; Figure 1). There was suggestive evidence of later pubertal onset (TV) with increasing PCDF or co-PCB concentrations but not with PCDF TEQs, co-PCB TEQs, or ΣPCB levels (Table 3; Figure 1). Similar findings were observed in adjusted interval-censored models. For example, TCDD was associated with later onset (approximately 5.5 months) (TV) for the highest compared with the lowest quartile of serum levels (95% CI, –0.6 to 11.9; *p*-trend = 0.07) [see Supplemental Material, Table 1 (http://dx.doi.org/10.1289/ehp.1003102)].

**Table 3:** Adjusted HRs^*a*^ and 95% CIs for associations of quartiles of serum dioxins, furans, and PCBs with pubertal onset between ages 8 and 12 years among Chapaevsk boys (*n* = 453).

Adjusted HR (95% CI)						
TEQ measures			Concentration measures		
Organochlorine quartiles (Q)	TV > 3 mL*b*	G2+*b*	TV > 3 mL*b*	G2+*b*
Total TEQ (pg TEQ/g lipid)								
Q1 (< 14)		1.00		1.00		NA*c*		NA*c*
Q2 (14 to < 20)		1.03 (0.72–1.47)		0.80 (0.58–1.11)				
Q3 (20 to < 30)		0.95 (0.57–1.35)		0.90 (0.66–1.23)				
Q4 (30 to 175)		0.81 (0.58–1.15)		0.91 (0.67–1.23)				
*p*-trend		0.19		0.77				
TCDD (pg TEQ/g lipid)*d*						TCDD (pg/g lipid)		
Q1 (< 1.3)						1.00		1.00
Q2 (1.3–2.7)						0.97 (0.70–1.34)		0.99 (0.73–1.34)
Q3 (2.8–3.9)						0.89 (0.63–1.24)		1.03 (0.76–1.40)
Q4 (4.0–45)						0.69 (0.48–0.98)		1.08 (0.79–1.48)
*p*-trend						0.04		0.60
PCDD TEQ (pg TEQ/g lipid)						PCDD (pg/g lipid)		
Q1 (< 5)		1.00		1.00		1.00		1.00
Q2 (5–7.9)		0.87 (0.62–1.21)		0.75 (0.55–1.03)		0.93 (0.67–1.31)		0.92 (0.68–1.26)
Q3 (8–12.9)		0.61 (0.43–0.85)		0.76 (0.56–1.03)		0.88 (0.63–1.23)		1.06 (0.78–1.43)
Q4 (13–90)		0.68 (0.49–0.95)		0.92 (0.69–1.25)		0.70 (0.50–1.00)		1.02 (0.74–1.39)
*p*-trend		0.006		0.64		0.05		0.70
PCDF TEQ (pg TEQ/g lipid)						PCDF (pg/g lipid)		
Q1 (< 3)		1.00		1.00		1.00		1.00
Q2 (3–3.9)		1.21 (0.85–1.72)		0.85 (0.62–1.18)		0.91 (0.65–1.27)		0.90 (.067–1.21)
Q3 (4–6.9)		1.08 (0.77–1.52)		1.06 (0.79–1.44)		0.88 (0.63–1.23)		0.93 (0.69–1.26)
Q4 (7–154)		0.86 (0.60–1.24)		0.80 (0.58–1.12)		0.75 (0.53–1.06)		0.89 (0.65–1.21)
*p*-trend		0.32		0.44		0.11		0.51
Co-PCB TEQ (pg TEQ/g lipid)						Co-PCB (pg/g lipid)		
Q1 (< 4.5)		1.00		1.00		1.00		1.00
Q2 (4.5–6.4)		1.23 (0.88–1.70)		1.14 (0.85–1.54)		1.13 (0.80–1.57)		1.04 (0.77–1.42)
Q3 (6.5–9.4)		1.12 (0.79–1.59)		0.97 (0.71–1.33)		0.85 (0.60–1.19)		0.85 (0.63–1.16)
Q4 (9.5–67)		1.02 (0.72–1.43)		1.02 (0.75–1.39)		0.78 (0.67–1.10)		0.87 (0.64–1.18)
*p*-trend		0.88		0.84		0.08		0.24
ΣPCBs (ng/g lipid)*e*						ΣPCBs (ng/g lipid)*e*		
Q1 (< 175)		NA*c*		NA*c*		1.00		1.00
Q2 (175 to < 250)						1.19 (0.84–1.69)		1.01 (0.74–1.39)
Q3 (250 to < 400)						1.12 (0.79–1.58)		1.14 (0.84–1.55)
Q4 (400 to 4,248)						0.97 (0.68–1.39)		1.14 (0.83–1.55)
*p*-trend						0.79		0.34
**a**Adjusted for birth weight, gestational age, parental education, household income, diet at 8 or 9 years old (total calories, percent protein, percent fat), blood lead ≥ 5 μg/dL, maternal pregnancy alcohol intake, baseline height, and BMI. **b**TV > 3 mL; G2+. **c**Concentration or TEQ measures not applicable (NA). **d**TCDD TEQ is identical to TCDD concentration. **e***n* = 448.								

In sensitivity analyses, the observed associations of dioxins with later pubertal onset by TV criteria were similar in GEE models and, in Cox models, were essentially unchanged after inclusion of maternal age at menarche, excluding boys who were pubertal at enrollment, excluding twins and siblings, or adjusting for year of serum analyses. However, adjustment for enrollment height and BMI attenuated the findings. For example, for onset by TV, the fourth (vs. first) quartile HR for total TEQs or PCDF TEQs, respectively, were 0.75 (95% CI, 0.54–1.05; *p*-trend = 0.08) and 0.76 (95% CI, 0.54–1.09; *p*-trend = 0.12) without height and BMI adjustment compared with 0.81 (95% CI, 0.58–1.15; *p*-trend = 0.19) and 0.86 (95% CI, 0.60–1.24; *p*-trend = 0.32) with adjustment (Table 3).

Adjusting models for ΣPCB levels resulted in slightly stronger associations of dioxin-like measures with later onset by TV [see Supplemental Material, Table 2 (http://dx.doi.org/10.1289/ehp.1003102)]. For example, the fourth (vs. first) quartile HR for total TEQs was 0.63 (95% CI, 0.38– 1.06; *p*-trend = 0.07) compared with 0.81 (95% CI, 0.58–1.15; *p*-trend = 0.19) without adjustment for ΣPCBs. Although the association of dioxin-like exposure measures with onset by G2+ remained largely nonsignificant, these associations were also stronger for most exposure measures after adjustment for ΣPCB levels (see Supplemental Material, Table 2). For example, the fourth (vs. first) quartile HR for total TEQs was 0.66 (95% CI, 0.41–1.05; *p*-trend = 0.12) compared with 0.91 (95% CI, 0.67–1.23; *p*-trend = 0.77) without ΣPCB adjustment.

ΣPCB levels were not associated with pubertal onset in the primary study analyses (Table 3), but secondary analyses, although mostly nonsignificant, supported a tendency for earlier pubertal onset by both TV and G2+ criteria with increasing ΣPCBs in models adjusted for dioxin-like measures [see Supplemental Material, Table 3 (http://dx.doi.org/10.1289/ehp.1003102)]. For example, the fourth (vs. first) quartile HR for ΣPCBs (adjusted for total TEQs) was 1.41 (95% CI, 0.82–2.42; *p*-trend = 0.23) for onset by TV and 1.51 (95% CI, 0.94–2.43; *p*-trend = 0.08) for onset by G2+.

## Discussion

This is perhaps the only large prospective cohort study of the relation of serum peripubertal dioxins, furans, and PCBs with physician-assessed male pubertal onset. The results demonstrate a relation of peripubertal dioxin exposure measures with subsequent delays in testicular maturation. Study results are consistent with animal models in which delayed male pubertal onset, assessed by genital maturation, is a consistently demonstrable correlate of early-life dioxin exposure (Bell et al. 2007; Hamm et al. 2003; Theobald et al. 2003).

In contrast, findings have been inconsistent among previous epidemiologic studies that examined late pubertal milestones rather than pubertal onset (potentially a more sensitive end point) and have been limited either by small sample size, cross-sectional design, self-reported pubertal staging, or lack of an exposure biomarker. For example, 80 Belgian teenage boys living near an incinerator (a presumed dioxin exposure source) had later sexual maturity, including smaller TV, compared with boys in an unpolluted town; however, TV did not correlate with indirect [chemical-activated luciferase gene expression (CALUX) assay] measures of dioxin exposure (Den Hond et al. 2002). Conversely, in a large (*n* = 887) cross-sectional study of 14- to 15-year-old Belgian boys, serum organochlorine levels [PCBs, *p*,*p´*-DDE (dichlorodiphenyldichloroethylene), and hexachlorobenzene] were associated with earlier genital development (increased odds of G3) on routine school health examinations performed, on average, within about 1 month of serum collection (Den Hond et al. 2010). Biomarkers of dioxin exposure were not assessed, but the PCB findings are consistent with our secondary analyses suggestive of possible earlier pubertal onset with increasing PCB exposures [Supplemental Material, Table 3 (http://dx.doi.org/10.1289/ehp.1003102)]. Among 14- to 18-year-old Dutch boys, pubertal maturation, including TV, was not associated with perinatal or concurrent dioxin levels. There were only 15 boys in this study, however, and as with the Belgian studies, assessments focused on late stages of puberty (Leijs et al. 2008). Finally, among 244 (primarily 12- to 14-year-old) North Carolina boys, age of self-reported pubertal stages was not associated with measures of prenatal PCB or DDE exposure (Gladen et al. 2000). Although larger than most other studies, the North Carolina assessments did not include measures of dioxin, TV, or pubertal onset.

Several potential limitations may affect interpretation of study findings. First, although both TV and G2+ maturation reflect hypothalamic–pituitary–gonadal (HPG) activation, associations were observed for TV but not genital staging (Table 3), with suggestive but mostly nonsignificant G2+ delays observed in association with dioxin-like measures only after adjustment for ΣPCBs. Assessment of TV by palpation and comparison with a standardized orchidometer is considered a more precise measure of gonadal development and pubertal status than genital staging (Biro et al. 1995; Euling et al. 2008) with minimal intraobserver variability (Carlsen et al. 2000), which could account for its greater sensitivity. In addition, testicular growth reflects both luteinizing hormone and follicle-stimulating hormone stimulation and paracrine androgen actions, whereas penile and scrotal maturation (G2) are primarily influenced by circulating androgens (Macleod et al. 2010; Raivio et al. 2007). In animal models, early-life dioxin exposure inhibits androgen biosynthesis and disrupts the HPG axis (Clements et al. 2009; Cooke et al. 1998; Fukuzawa et al. 2004; Kakeyama et al. 2008). If dioxins disproportionately impair gonadotropin secretion relative to androgen biosynthesis, TV could be affected more than G2.

Although the mean age of pubertal onset of boys by TV (10.5 years) is consistent with other studies (Herman-Giddens et al. 2001; Susman et al. 2010), later onset has been observed in Danish boys (Sorensen et al. 2010) and mean age of onset in Chapaevsk boys by G2 (9.4 years) is younger than reported elsewhere. Whether the apparent earlier G2 reflects a true difference between Chapaevsk boys and other populations is unclear, because most data on male pubertal development are cross-sectional and/or collected at older ages (Biro et al. 1995; Sun et al. 2005; Susman et al. 2010), making comparisons difficult. However, given that study exams were all performed by a single physician, internal comparisons among study boys should be valid and unbiased.

Additional study limitations include the observation that a number of Chapaevsk boys had entered puberty before study enrollment (e.g., 12% of 8-year-olds and 18% of 9-year-olds by TV); it is unclear whether exposure measures obtained after pubertal onset reflect relevant exposure risk. However, the relationship of dioxins with TV persisted after exclusion of boys in puberty at enrollment. Although lower BMI and socioeconomic status were associated with both higher serum dioxins and later pubertal onset in this cohort (Burns et al. 2009) and thus may confound results, the relationship of dioxins with TV persisted after adjustment for height, BMI, and measures of socioeconomic status (Table 3).

Finally, if dioxin-associated alterations in male pubertal development are aryl hydrocarbon receptor (AhR)–mediated toxicities (Theobald et al. 2003), then the apparent stronger association of TV with PCDD TEQs compared with PCDF or co-PCB TEQs is difficult to explain. It is possible that non-AhR-mediated mechanisms are relevant (Butler et al. 2004) and that congeners contributing to such mechanisms correlate better with some TEQ measures than others. In addition, sources of environmental contamination with PCDDs, PCDFs, or PCBs may vary, as the latter are a manufactured product rather than a by-product of chemical production or incineration. Thus, confounding by differing unmeasured co-occurring exposures may explain apparent differential TEQ effects. The results of secondary analyses suggestive of earlier, rather than later, puberty in association with non-co-planar PCB exposures are consistent with the possibility that non-AhR-related mechanisms may be important to organochlorine-associated alterations in male pubertal onset. Although Taiwanese boys with substantial prenatal furan and PCB exposure had no apparent exposure-associated differences in pubertal stage (Hsu et al. 2005), concurrent PCB levels have been associated with earlier, not later, male genital development in other populations (Den Hond et al. 2010).

## Conclusions

Although recent emphasis has been placed on environmental risk factors for earlier breast development and menarche in girls, environmental contaminants may also delay puberty (Selevan et al. 2003; Wu et al. 2003) and impact puberty in boys. In this study, serum dioxins measured at age 8 or 9 years were associated with later male pubertal onset (by TV criteria). Although this was not indicative of clinically delayed onset, modest changes in the mean value of a health indicator, such as pubertal onset, can signal substantial changes in the prevalence of clinically evident disease within a population (Korrick and Bellinger 2007).

## Supplemental Material

(132 KB) PDF

## References

[r1] Barr JR, Maggio VL, Barr DB, Turner WE, Sjodin A, Sandau CD (2003). New high-resolution mass spectrometric approach for the measurement of polychlorinated biphenyls and organochlorine pesticides in human serum.. J Chromatogr B.

[r2] Bell DR, Clode S, Fan MQ, Fernandes A, Foster PM, Jiang T (2007). Toxicity of 2,3,7,8-tetrachlorodibenzo-*p*-dioxin in the developing male Wistar(Han) rat II: chronic dosing causes developmental delay.. Toxicol Sci.

[r3] Biro FM, Galvez MP, Greenspan LC, Succop PA, Vangeepuram N, Pinney SM (2010). Pubertal assessment method and baseline characteristics in a mixed longitudinal study of girls.. Pediatrics.

[r4] Biro FM, Lucky AW, Huster G, Morrison JA (1995). Pubertal staging in boys.. J Pediatr.

[r5] Biro FM, Lucky AW, Simbartl LA, Barton BA, Daniels SR, Striegel-Moore R (2003). Pubertal maturation in girls and the relationship to anthropometric changes: pathways through puberty.. J Pediatr.

[r6] Burns JS, Williams PL, Sergeyev O, Korrick S, Lee MM, Revich B (2009). Predictors of serum dioxins and PCBs among peripubertal Russian boys.. Environ Health Perspect.

[r7] Butler RA, Kelley ML, Olberding KE, Gardner GR, Van Beneden RJ (2004). Aryl hydrocarbon receptor (AhR)-independent effects of 2,3,7,8-tetrachlorodibenzo-*p*-dioxin (TCDD) on softshell clam (*Mya arenaria*) reproductive tissue.. Comp Biochem Physiol C Toxicol Pharmacol.

[r8] Carlsen E, Andersen AG, Buchreitz L, Jorgensen N, Magnus O, Matulevicuus V (2000). Inter-observer variation in the results of the clinical andrological examination including estimation of testicular size.. Int J Androl.

[r9] Clements RJ, Lawrence RC, Blank JL (2009). Effects of intrauterine 2,3,7,8-tetrachlorodibenzo-*p*-dioxin on the development and function of the gonadotropin releasing hormonal neuronal system in the male rat.. Reprod Toxicol.

[r10] Cooke GM, Price CA, Oko RJ (1998). Effects of *in utero* and lactational exposure to 2,3,7,8-terachlorodibenzo-*p*-dioxin (TCDD) on serum androgens and steroidogenic enzyme activities in the male rat reproductive tract.. J Steroid Biochem Molec Biol.

[r11] de Onis M, Onyango AW, Borghi E, Siyam A, Nishida C, Siekmann J (2007). Development of a WHO growth reference for school-aged children and adolescents.. Bull WHO.

[r12] Den Hond E, Dhooge W, Bruckers L, Shoeters G, Nelen V, van de Mieroop E (2010). Internal exposure to pollutants and sexual maturation in Flemish adolescents.. J Expo Sci Environ Epidemiol.

[r13] Den Hond E, Roels HA, Hoppenbrouwers K, Nawrot T, Thijs L, Vandermeulen C (2002). Sexual maturation in relation to polychlorinated aromatic hydrocarbons: Sharpe and Skakkebaek’s hypothesis revisited.. Environ Health Perspect.

[r14] Euling SY, Herman-Giddens ME, Lee PA, Selevan SG, Juul A, Sorensen TIA (2008). Examination of U.S. puberty-timing data from 1940 to 1994 for secular trends: panel findings.. Pediatrics.

[r15] Finkelstein JS, Klibanski A, Neer RM (1996). A longitudinal evaluation of bone mineral density in adult men with histories of delayed puberty.. J Clin Endocrinol Metab.

[r16] Fukuzawa NH, Ohsako S, Wu Q, Sakaue M, Fujii-Kuriyama Y, Baba T (2004). Testicular cytochrome P450scc and LHR as possible targets of 2,3,7,8-tetrachlorodibenzo-*p-*dioxin (TCDD) in the mouse.. Mol Cell Endocrinol.

[r17] Gladen BC, Ragan B, Rogan WJ (2000). Pubertal growth and development and prenatal and lactational exposure to polychlorinated biphenyls and dichlorodiphenyl dichloroethene.. J Pediatr.

[r18] Graber JA, Seelely JR, Brooks-Gunn J, Lewinsohn PM (2004). Is pubertal timing associated with psychopathology in young adulthood.. J Am Acad Child Adolesc Psychiatry.

[r19] Hamm JT, Chen CY, Birnbaum LS (2003). A mixture of dioxins, furans, and non-*ortho* PCBs based upon consensus toxic equivalency factors produces dioxin-like reproductive effects.. Toxicol Sci.

[r20] Hauser R, Sergeyev O, Korrick S, Lee MM, Revich B, Gitin E (2008). Association of blood lead levels with onset of puberty in Russian boys.. Environ Health Perspect.

[r21] Herman-Giddens ME, Slora EJ, Wasserman RC, Bourdony CJ, Bhapkar MV, Koch GG (1997). Secondary sexual characteristics and menses in young girls seen in office practice: a study from the Pediatric Research in Office Settings network.. Pediatrics.

[r22] Herman-Giddens ME, Wang L, Koch G (2001). Secondary sexual characteristics in boys: estimates from the National Health and Nutrition Examination Survey III, 1988–1994.. Arch Pediatr Adolesc Med.

[r23] Hsu PC, Lai TJ, Guo NW, Lambert GH, Guo YL (2005). Serum hormones in boys prenatally exposed to polychlorinated biphenyls and dibenzofurans.. J Toxicol Environ Health, Part A.

[r24] Jacobson-Dickman E, Lee MM (2009). The influence of endocrine disruptors on pubertal timing.. Curr Opin Endocrinol Diabetes Obes.

[r25] Kakeyama M, Sone H, Tohyama C. (2008). Perinatal exposure of female rats to 2,3,7,8-tetrachlorodibenzo-*p*-dioxin induces central precocious puberty in the offspring.. J Endocrinol.

[r26] Korrick SA, Bellinger DC (2007). Invited commentary: persistent organic pollutants and childhood learning and behavioral disorders.. J Epidemiol Community Health.

[r27] Leijs MM, Koppe JG, Olie K, van Aalderen WMC, de Voogt P, Vulsma T (2008). Delayed initiation of breast development in girls with higher prenatal dioxin exposure; a longitudinal cohort study.. Chemosphere.

[r28] Link B, Gabrio T, Zoellner I, Piechotowski I, Paepke O, Herrmann T (2005). Biomonitoring of persistent organochlorine pesticides, PCDD/PCDFs and dioxin-like PCBs in blood of children from southwest Germany (Baden-Wuerttemberg) from 1993–2003.. Chemosphere.

[r29] Macleod DJ, Sharpe RM, Welsh M, Fisken M, Scott HM, Hutchison GR (2010). Androgen action in the masculinization programming window and development of male reproductive organs.. Int J Androl.

[r30] Martinchik AN, Baturin AK, Baeva VS, Feoktistova AI, Piatnitskaia IN, Azizbekian BA (1998). Development of a method of studying actual nutrition according to analysis of the frequency of consumption of food products: creation of a questionnaire and general evaluation of the reliability of the method. Vopr Pitan.

[r31] Michaud PA, Suris JC, Deppen A (2006). Gender-related psychological and behavioural correlates of pubertal timing in a national sample of Swiss adolescents.. Mol Cell Endocrinol.

[r32] Patterson DG, Hampton L, Lapeza CR, Belser WT, Green V, Alexander L (1987). High-resolution gas chromatographic/high-resolution mass spectroscopic analysis of human serum on a whole-weight and lipid basis for 2,3,7,8-tetrachlorodibenzo-*p*-dioxin.. Anal Chem.

[r33] Phillips DL, Pirkle JL, Burse VW, Bernert JT, Henderson LO, Needham LL (1989). Chlorinated hydrocarbon levels in human serum: effects of fasting and feeding.. Arch Environ Contam Toxicol.

[r34] Raivio T, Wikstrom AM, Dunkel L (2007). Treatment of gonadotropin-deficient boys with recombinant human FSH: long-term observation and outcome.. Eur J Endocrinol.

[r35] Revich B, Brodsky E, Sotskov Y. (1999). Dioxin in environmental, blood, breast milk, cow milk in Chapaevsk town.. Organohalogen Compounds.

[r36] Schecter A, Cramer P, Boggess K, Stanley J, Papke O, Olson J (2001). Intake of dioxins and related compounds from food in the U.S. population.. J Toxicol Environ Health A.

[r37] Schoeters G, Den Hond E, Dhooge W, van Larebeke N, Leijs M. (2008). Endocrine disruptors and abnormalities of pubertal development.. Basic Clin Pharmacol Toxicol.

[r38] Selevan SG, Rice DC, Hogan KA, Euling SLY, Pfahles-Hutchens A, Bethel J (2003). Blood lead concentration and delayed puberty in girls.. N Engl J Med.

[r39] Sergeyev O, Saharov I, Shelepchikov AA, Revich B, Sotskov Y, Brodsky E (2007). Levels of PCDDs/PCDFs in the environment and food after 3 years of full plant inactivity, Chapaevsk, Russia.. Organohalogen Compd.

[r40] Sjodin A, McGahee EE, Focant JF, Jones RS, Lapeza CR, Zhang Y (2004). Semiautomated high-throughput extraction and cleanup method for the measurement of polybrominated diphenyl ethers, polybrominated biphenyls, and polychlorinated biphenyls in human serum.. Anal Chem.

[r41] Sorensen K, Aksglaede L, Holm Petersen J, Juul A. (2010). Recent changes in pubertal timing in healthy Danish boys: associations with body mass index.. J Clin Endocrinol Metab.

[r42] Sun SS, Schubert CM, Liang R, Roche AF, Kulin HE, Lee PA (2005). Is sexual maturity occurring earlier among U.S. children?. J Adolesc Health.

[r43] Susman EJ, Houts RM, Steinberg L, Belsky J, Cauffman E, DeHart G (2010). Longitudinal development of secondary sexual characteristics in girls and boys between ages 9½ and 15½ years.. Arch Pediatr Adolesc Med.

[r44] Tanner JM, Whitehouse RH (1976). Clinical longitudinal standards for height, weight, height velocity, weight velocity, and stages of puberty.. Arch Dis Child.

[r45] Theobald HM, Kimmel GL, Peterson RE (2003). Developmental and reproductive toxicity of dioxins and related chemicals. In: Dioxins and Health (Schechter A, Gasiewicz TA, eds). 2nd ed.

[r46] Turner W, DiPeitro E, Lapeza C, Green V, Gill J, Patterson DG (1997). A fast universal automated cleanup system for the isotope-dilution high-resolution mass spectrometric analysis of PCDDs, PCDFs, coplanar PCBs, PCB congeners, and persistent pesticides from the same serum sample.. Organohalogen Compd.

[r47] Van den Berg M, Birnbaum LS, Denison M, De Vito M, Farland W, Feeley M (2006). Review: the 2005 World Health Organization reevaluation of human and mammalian toxic equivalency factors for dioxins and dioxin-like compounds.. Toxicol Sci.

[r48] Van Lenthe FJ, Kemper CG, van Mechelen W (1996). Rapid maturation in adolescence results in greater obesity in adulthood: the Amsterdam Growth and Health Study.. Am J Clin Nutr.

[r49] Williams PL, Sergeyev O, Lee MM, Korrick SA, Burns JS, Humblet O (2010). Blood lead levels and delayed onset of puberty in a longitudinal study of Russian boys.. Pediatrics.

[r50] Wu T, Buck GM, Mendola P (2003). Blood lead levels and sexual maturation in U.S. girls: The Third National Health and Nutrition Examination Survey, 1988–1994.. Environ Health Perspect.

