# Supplementary material for: Dioxin Exposure and Age of Pubertal Onset among Russian Boys
Source: Environ Health Perspect. 2011 Apr 28;119(9):1339–44. doi: 10.1289/ehp.1003102 (PMC3230396; doi:10.1289/ehp.1003102)
Supplement: (132 KB) PDF [file ehp.1003102.s001.pdf]

Supplemental Material:

Dioxin Exposure and Age of Pubertal Onset Among Russian Boys

Susan A. Korrick, Mary M. Lee, Paige L. Williams, Oleg Sergeyev, Jane S. Burns, Donald G. Patterson Jr., Wayman E. Turner, Larry L. Needham, Larisa Altshul, Boris Revich, Russ Hauser

Supplemental Material, Table 1: Adjusted[a] change in mean age at pubertal onset (months) and 95% confidence intervals by quartiles of serum dioxins, furans, and PCBs among Chapaevsk boys (n=453)

Adjusted Change in Mean Age at Onset in Months (95% Confidence Interval)				
Organochlorine Quartiles	Toxic Equivalent Measures		Concentration Measures	
	**TV>3ml[b]**	**G2+[b]**	**TV>3ml[b]**	**G2+[b]**
Total TEQ (pg TEQ/g lipid)				
Q1 (< 14)	Ref.	Ref.	N/A[c]	N/A[c]
Q2 (14 -<20)	-0.9 (-7.2, 5.5)	4.5 (-2.0, 11.0)		
Q3 (20 -<30)	0.7 (-5.5, 7.0)	2.0 (-4.5, 8.4)		
Q4 (30 - 175)	3.3 (-2.7, 9.4)	2.5 (-3.8, 8.7)		
p- trend	*0.22*	*0.65*		
TCDD (pg TEQ/g lipid)[d]			TCDD (pg/g lipid)	
Q1 (< 1.3)			Ref.	Ref.
Q2 (1.3 - 2.7)			1.2 (-4.6, 7.1)	0.1 (-6.0, 6.2)
Q3 (2.8 - 3.9)			2.5 (-3.5, 8.5)	-0.7 (-6.9, 5.5)
Q4 (4.0 - 45)			5.7 (-0.6, 11.9)	0.0 (-6.4, 6.4)
p-trend			*0.07*	*0.94*
PCDD TEQ (pg TEQ/g lipid)			PCDD (pg/g lipid)	
Q1 (< 5)	Ref.	Ref.	Ref.	Ref.
Q2 (5 - 7.9)	3.5 (-2.6, 9.6)	6.3 (0.0, 12.6)	1.7 (-4.3, 7.6)	1.4 (-4.9, 7.6)
Q3 (8 - 12.9)	8.0 (2.1, 13.8)	6.6 (0.5, 12.7)	1.5 (-4.4, 7.5)	-1.0 (-7.3, 5.2)
Q4 (13 - 90)	6.7 (0.8, 12.6)	4.0 (-2.1, 10.1)	7.3 (1.2, 13.4)	-0.2 (-6.5, 6.1)
p-trend	*0.01*	*0.21*	*0.03*	*0.77*
PCDF TEQ (pg TEQ/g lipid)			PCDF (pg/g lipid)	
Q1 (< 3)	Ref.	Ref.	Ref.	Ref.
Q2 (3 - 3.9)	-3.3 (-9.5, 2.9)	3.6 (-2.8, 10.0)	4.0 (-1.9, 9.8)	3.9 (-2.1, 9.9)
Q3 (4 - 6.9)	-1.6 (-7.6, 4.4)	-0.9 (-7.1, 5.3)	3.3 (-2.6, 9.2)	1.0 (-5.1, 7.2)
Q4 (7 - 154)	2.4 (-3.9, 8.8)	4.7 (-1.7, 11.2)	6.6 (0.6, 12.6)	2.8 (-3.4, 9.0)
p- trend	*0.38*	*0.38*	*0.05*	*0.54*
co-PCB TEQ (pg TEQ/g lipid)			co-PCB (pg/g lipid)	
Q1 (<4.5)	Ref.	Ref.	Ref.	Ref.
Q2 (4.5 - 6.4)	-4.7 (-10.6, 1.1)	-3.8 (-9.8, 2.2)	-3.6 (-9.5, 2.3)	-2.5 (-8.6, 3.6)
Q3 (6.5 - 9.4)	-2.1 (-8.3, 4.1)	0.1 (-6.2, 6.4)	1.8 (-4.1, 7.7)	1.6 (-4.5, 7.7)
Q4 (9.5 - 67)	-1.0 (-7.1, 5.1)	-1.6 (-7.8, 4.6)	3.6 (-2.4, 9.6)	0.4 (-5.8, 6.7)
p-trend	*0.97*	*0.92*	*0.11*	*0.61*
ΣPCBs (ng/g lipid)[e]			ΣPCBs (ng/g lipid)[e]	
Q1 (< 175)	N/A[c]	N/A[c]	Ref.	Ref.
Q2 (175 -<250)			-4.7 (-10.9, 1.5)	-1.6 (-7.9, 4.7)
Q3 (250 -<400)			-2.9 (-9.0, 3.2)	-3.0 (-9.2, 3.3)
Q4 (400 - 4248)			-1.4 (-7.6, 4.8)	-3.7 (-10.1, 2.7)
p-trend			*0.79*	*0.23*

Supplemental Material, Table 1 (continued):

[a]Adjusted for: birth weight, gestational age, parental education, household income, 8/9-year diet (total calories, %protein, %fat), blood lead ≥5 μg/dl, maternal pregnancy alcohol intake, baseline height and BMI.

[b]Testicular volume (TV)>3ml; genitalia staging ≥2 (G2+).

[c]Concentration or TEQ measures not applicable.

[d]TCDD TEQ is identical to TCDD concentration.

[e]n=448.

Supplemental Material, Table 2: Adjusted (*including adjustment for non-co-planar PCBs*) hazard ratios (HRs)[a] and 95% confidence intervals for associations of serum dioxins, furans , and co-planar PCBs with pubertal onset between ages 8-12 years among Chapaevsk boys (n=448)

	Adjusted HR (95% Confidence Interval)			
Organochlorine Quartiles	Toxic Equivalent Measures (adjusted for ΣPCB quartiles)		Concentration Measures (adjusted for ΣPCB quartiles)	
	**TV>3ml[b]**	**G2+[b]**	**TV>3ml[b]**	**G2+[b]**
Total TEQ (pg TEQ/g lipid)				
Q1 (< 14)	1.00	1.00	N/A[c]	N/A[c]
Q2 (14 -<20)	0.88 (0.59, 1.32)	0.71 (0.50, 1.02)		
Q3 (20 -<30)	0.75 (0.48, 1.18)	0.70 (0.46, 1.06)		
Q4 (30 - 175)	0.63 (0.38, 1.06)	0.66 (0.41, 1.05)		
p- trend	*0.07*	*0.12*		
TCDD (pg TEQ/g lipid)[d]			TCDD (pg/g lipid)	
Q1 (< 1.3)			1.00	1.00
Q2 (1.3 - 2.7)			0.94 (0.68, 1.32)	1.00 (0.73, 1.36)
Q3 (2.8 - 3.9)			0.80 (0.56, 1.15)	0.98 (0.71, 1.35)
Q4 (4.0 - 45)			0.62 (0.41, 0.93)	0.99 (0.69, 1.44)
p-trend			*0.02*	*0.94*
PCDD TEQ (pg TEQ/g lipid)			PCDD (pg/g lipid)	
Q1 (< 5)	1.00	1.00	1.00	1.00
Q2 (5 - 7.9)	0.78 (0.55, 1.11)	0.75 (0.55, 1.04)	0.88 (0.62, 1.25)	0.91 (0.66, 1.27)
Q3 (8 - 12.9)	0.51 (0.35, 0.73)	0.71 (0.51, 0.98)	0.84 (0.58, 1.21)	1.02 (0.74, 1.43)
Q4 (13 - 90)	0.59 (0.39, 0.90)	0.84 (0.59, 1.21)	0.64 (0.43, 0.95)	0.96 (0.68, 1.37)
p-trend	*0.001*	*0.22*	*0.03*	*0.98*
PCDF TEQ (pg TEQ/g lipid)			PCDF (pg/g lipid)	
Q1 (< 3)	1.00	1.00	1.00	1.00
Q2 (3 - 3.9)	1.14 (0.78, 1.65)	0.79 (0.55, 1.11)	0.84 (0.59, 1.19)	0.84 (0.61, 1.15)
Q3 (4 - 6.9)	0.96 (0.64, 1.46)	0.89 (0.61, 1.30)	0.80 (0.54, 1.17)	0.83 (0.58, 1.18)
Q4 (7 - 154)	0.79 (0.49, 1.26)	0.61 (0.40, 0.96)	0.64 (0.41, 0.99)	0.73 (0.49, 1.08)
p- trend	*0.25*	*0.06*	*0.05*	*0.13*
co-PCB TEQ (pg TEQ/g lipid)			co-PCB (pg/g lipid)	
Q1 (<4.5)	1.00	1.00	1.00	1.00
Q2 (4.5 - 6.4)	1.20 (0.85, 1.60)	1.07 (0.78, 1.47)	1.09 (0.77, 1.52)	0.98 (0.71, 1.34)
Q3 (6.5 - 9.4)	1.14 (0.77, 1.71)	0.85 (0.59, 1.23)	0.74 (0.51, 1.08)	0.73 (0.52, 1.03)
Q4 (9.5 - 67)	1.02 (0.64, 1.62)	0.84 (0.55, 1.28)	0.68 (0.44, 1.04)	0.67 (0.46, 0.98)
p-trend	*0.91*	*0.27*	*0.05*	*0.02*

[a]Adjusted for: birth weight, gestational age, parental education, household income, 8/9-year diet (total calories, %protein, %fat), blood lead ≥5 μg/dl, maternal pregnancy alcohol intake, baseline height and BMI, and non-co-planar PCBs (ΣPCBs).

[b]Testicular volume (TV)>3ml; genitalia staging ≥2 (G2+).

[c]Concentration or TEQ measures not applicable.

[d]TCDD TEQ is identical to TCDD concentration.

Supplemental Material, Table 3: Adjusted hazard ratios (HRs)[a] and 95% confidence intervals for associations of serum ΣPCBs with pubertal onset between ages 8-12 years among Chapaevsk boys (including adjustment for dioxins, furans, and co-planar PCBs, n=448)

	Adjusted HR (95% Confidence Interval)			
ΣPCB Quartiles (ng/g lipid)	Adjusted for Toxic Equivalent Measures (pg TEQ/g lipid)		Adjusted for Concentration Measures (pg/g lipid)	
	**TV>3ml[b]**	**G2+[b]**	**TV>3ml[b]**	**G2+[b]**
ΣPCBs (+adjusted Total TEQ)				
Q1 (< 175)	1.00	1.00	N/A[c]	N/A[c]
Q2 (175 -<250)	1.36 (0.91, 2.04)	1.21 (0.84, 1.74)		
Q3 (250 -<400)	1.41 (0.90, 2.22)	1.46 (0.96, 2.21)		
Q4 (400 - 4248)	1.41 (0.82, 2.42)	1.51 (0.94, 2.43)		
p- trend	*0.23*	*0.08*		
ΣPCBs (+adjusted TCDD TEQ)[d]			(+adjusted TCDD concentration)	
Q1 (< 175)			1.00	1.00
Q2 (175 -<250)			1.26 (0.88, 1.79)	1.01 (0.74, 1.40)
Q3 (250 -<400)			1.33 (0.91, 1.92)	1.15 (0.81, 1.61)
Q4 (400 - 4248)			1.27 (0.85, 1.92)	1.14 (0.80, 1.64)
p-trend			*0.23*	*0.42*
ΣPCBs (+adjusted PCDD TEQ)			(+adjusted PCDD concentration)	
Q1 (< 175)	1.00	1.00	1.00	1.00
Q2 (175 -<250)	1.45 (1.01, 2.09)	1.10 (0.80, 1.52)	1.25 (0.87, 1.79)	1.02 (0.73, 1.42)
Q3 (250 -<400)	1.51 (1.03, 2.21)	1.26 (0.90, 1.76)	1.25 (0.86, 1.80)	1.14 (0.82, 1.58)
Q4 (400 - 4248)	1.34 (0.87, 2.07)	1.19 (0.81, 1.73)	1.18 (0.79, 1.75)	1.15 (0.81, 1.62)
p-trend	*0.15*	*0.28*	*0.45*	*0.82*
ΣPCBs (+adjusted PCDF TEQ)			(+adjusted PCDF concentration)	
Q1 (< 175)	1.00	1.00	1.00	1.00
Q2 (175 -<250)	1.22 (0.83, 1.79)	1.13 (0.79, 1.60)	1.32 (0.91, 1.91)	1.11 (0.79, 1.55)
Q3 (250 -<400)	1.23 (0.81, 1.85)	1.31 (0.90, 1.92)	1.30 (0.87, 1.94)	1.27 (0.89, 1.82)
Q4 (400 - 4248)	1.17 (0.73, 1.88)	1.48 (0.96, 2.27)	1.29 (0.82, 2.03)	1.38 (0.92, 2.06)
p- trend	*0.57*	*0.06*	*0.31*	*0.09*
ΣPCBs (+adjusted co-PCB TEQ)			(+adjusted co-PCB concentration)	
Q1 (< 175)	1.00	1.00	1.00	1.00
Q2 (175 -<250)	1.15 (0.80, 1.66)	1.03 (0.74, 1.44)	1.31 (0.91, 1.87)	1.10 (0.79, 1.53)
Q3 (250 -<400)	1.08 (0.73, 1.61)	1.26 (0.87, 1.81)	1.36 (0.93, 2.00)	1.37 (0.97, 1.94)
Q4 (400 - 4248)	1.00 (0.61, 1.62)	1.33 (0.87, 2.03)	1.33 (0.85, 2.08)	1.50 (1.02, 2.22)
p-trend	*0.99*	*0.15*	*0.20*	*0.03*

[a]Adjusted for: birth weight, gestational age, parental education, household income, 8/9-year diet (total calories, %protein, %fat), blood lead ≥5 µg/dl, maternal pregnancy alcohol intake, baseline height and BMI, and dioxins, furans, or co-PCBs as specified above.

[b]Testicular volume (TV)>3ml; genitalia staging ≥2 (G2+).

[c]Concentration or TEQ measures not applicable.

[d]TCDD TEQ is identical to TCDD concentration.
